# Inter-individual variation in adaptations to endurance and resistance exercise training: genetic approaches towards understanding a complex phenotype

**DOI:** 10.1007/s00335-017-9732-5

**Published:** 2018-01-22

**Authors:** Heather L. Vellers, Steven R. Kleeberger, J. Timothy Lightfoot

**Affiliations:** 1 Immunity, Inflammation and, Disease Laboratory, National Institute of Environmental Health Sciences, 111 T.W. Alexander Dr., Building 101, E-224, Research Triangle Park, NC 27709 USA; 2 Department of Health and Kinesiology, Texas A&M University, College Station, TX 77843 USA

## Abstract

Exercise training which meets the recommendations set by the National Physical Activity Guidelines ensues a multitude of health benefits towards the prevention and treatment of various chronic diseases. However, not all individuals respond well to exercise training. That is, some individuals have no response, while others respond poorly. Genetic background is known to contribute to the inter-individual (human) and -strain (e.g., mice, rats) variation with acute exercise and exercise training, though to date, no specific genetic factors have been identified that explain the differential responses to exercise. In this review, we provide an overview of studies in human and animal models that have shown a significant contribution of genetics in acute exercise and exercise training-induced adaptations with standardized endurance and resistance training regimens, and further describe the genetic approaches which have been used to demonstrate such responses. Finally, our current understanding of the role of genetics and exercise is limited primarily to the nuclear genome, while only a limited focus has been given to a potential role of the mitochondrial genome and its interactions with the nuclear genome to predict the exercise training-induced phenotype(s) responses. We therefore discuss the mitochondrial genome and literature that suggests it may play a significant role, particularly through interactions with the nuclear genome, in the inherent ability to respond to exercise.

## Introduction

Regular daily exercise has multiple beneficial health outcomes including reductions in risk for cardiovascular disease (Manson et al. 1999), diabetes (LaMonte et al. 2005), several forms of cancer (Campbell and McTiernan 2007), stroke (Alevizos et al. 2005), neuro-cognitive dysfunctions (Verghese et al. 2003), all-cause mortality rates (Iestra et al. 2005), quality of life (Belardinelli et al. 1999), and lifespan (Moore et al. 2012; Paffenbarger et al. 1986). Such exercise-induced health benefits are due in part to solitary and combined effects of endurance and resistance training regimens which meet recommendations set by the National Physical Activity Guidelines Committee (2008). However, responses to exercise training are not consistent among all individuals, as some individuals respond well to training, while others respond poorly, even when accounting for factors including age, sex, and ethnic origin (Bouchard and Rankinen 2001; Bouchard et al. 1999). This known inter-individual variation in response to exercise training has primarily been characterized by aerobic capacity (VO_2max_) for endurance training (Bouchard et al. 1999; Pérusse et al. 2001), and by strength and muscle mass measurements for resistance training (Wilmore et al. 1998). With these measures, genetic background has been determined as a strong contributor to inter-individual variation in such measures of endurance and resistance training-induced adaptations. However, our current knowledge regarding links between genetics and inter-individual variation in response to exercise training is limited. The purpose of this review is to bring forth limitations in the literature, and discuss approaches that could be employed to circumvent such limitations.

One key limitation is the lack of a defined phenotype(s) for endurance and resistance training-induced responses, due largely to use of inconsistent research methods among investigators (i.e., modes, frequency, intensity, and duration of study protocols). Furthermore, it is becoming increasingly understood that phenotypes which define exercise training-induced adaptations are complex with numerous structural, functional, physiological, and molecular changes that occur in multiple organs (e.g., heart, skeletal muscle) and systems (cardiovascular, muscular) in response to exercise training. Thus, the complexity of physiological and molecular changes occurring with endurance and resistance training stresses the importance of establishing collaborative efforts among investigators which have expertise in the known phenotypic adaptations with endurance and resistance training.

Another limitation is identification of an appropriate animal modeling system that mimics heterogeneity in humans. Given the need to access specific tissues and organs (e.g., brain, heart) and the need to control confounding variables (e.g., diet, environment, background genetics/epigenetics), animal models remain necessary. The National Institutes of Health recognized this form of “bidirectional translation” (Rubio et al. 2010) as the ideal design for “a highly effective translational research program.” Indeed, multiple mammalian animal models have a long history of being used to understand basic principles of exercise physiology (e.g., Davis et al. 2014, Epp et al. 2006; Ferguson et al. 2014; Kao 1956; Poole et al. 2007; Poole and Erickson 2011; Roberts et al. 2014; Schaeffer et al. 1996) and responses to repeated exercise (e.g., Favier et al. 2016; Firshman et al. 2015; Kuster et al. 2014; Massett et al. 2015). These models are adequate to model exercise responses using molecular techniques; however, it is critical that the model simulates and controls the human genetic heterogeneity underlying variable responses to exercise so that conclusions derived are both translatable and generalizable.

The final section of this review will discuss briefly the mitochondrial genome and its interactions with the nuclear genome, and how different elements of the mitochondrial genome may contribute to the genetic regulation of exercise training adaptations. Mitochondria are cellular organelles responsible for sensing and initiating a multitude of signaling processes involved in energy metabolism, and the function of the mitochondria in eliciting such responses is largely dependent on the mitochondrial genome. Thus, since exercise is a metabolic stressor to the mitochondria, it is likely that the mitochondrial genome may contribute at least partially to the genetic regulation of exercise training-induced adaptations. To date, our primary understanding of genetics and exercise training-induced associations are studied from the nuclear genome, and with only a limited insight to the role of the mitochondrial genome. However, there are multiple aspects of the mitochondrial genome including heteroplasmy and copy number which could have significant influences on an individual’s response to exercise training.

## Animal models

For a variety of reasons, including ethical, research design, and systematic control concerns, it is difficult to use humans to examine exercise training alterations in many molecular systems as well as most organ systems. Thus, use of mammalian animal models provide a means to control for a wide variety of research design and environmental factors to investigate the genetic contribution to exercise training-induced adaptations, as well as allowing access to all organ systems. With animal models, however, genetic heterogeneity which mimics that of humans, is an important factor and is a concern for future studies that investigate underlying genetic contributors to exercise training adaptations. Several animal models mimic the heterogeneity of humans, though major limitations exist in most models that complicate the ability to identify underlying genetic factors. Herein, we discuss genetic models that have been used to evaluate the link between genetics and response to exercise training, and end with a discussion of relatively new models that could be used to translate findings between animals and humans.

### Inbred strains

Inbred strains (pigs, mice, and rats) provide cohorts of animals that are genetically homozygous, and thus provide the ability to interrogate specific genomes. Further, when multiple inbred strains are tested (i.e., “strain-screen”) for any phenotype, investigators then interrogate the amount of between-strain variation in characteristics and importantly, initial indications of the genomic location associated with that phenotype/trait [i.e., quantitative trait locus (QTL)]. Inbred mouse models have been used extensively to investigate inherent physical activity (Lightfoot et al. 2008, 2010), inherent aerobic capacity (Courtney and Massett 2012, 2014; Lightfoot et al. 2001, 2007), and inbred mouse and rat models have been developed for endurance exercise training (Massett et al. 2009, 2015; Massett and Berk 2005; Koch et al. 2013). However, a weakness of inbred models is the concern that since many of the classic inbred mouse and rat strains arose from common progenitor strains, large regions of the genomes from different inbred strains are actually the same [i.e., identity by descent (IBD)] and thus IBD issues prevent the examination of the full range of genomic variability with a particular trait. For example, within 40 classical inbred mouse lines, it is estimated that IBD ≈ 20% (Roberts et al. 2007; Yang et al. 2011) indicating at least 20% of the genome is not interrogated when using classical inbred strains. To our knowledge, no current estimates of IBD among rat inbred strains exist; however, a recent study compared two related rat inbred lines and estimated levels of IBD approaching 87% (Bell et al. 2011). Thus, higher IBD between inbred strains causes less genomic variation to be interrogated.

### Selectively bred animal model

The selectively bred animal model has been used extensively to investigate high-physical activity level in mice (Garland et al. 2011; Garland and Kelly 2006; Swallow et al. 1998b) and rats (Roberts et al. 2013, 2014), inherent endurance exercise capacity in rats (Barbato et al. 1998; Britton and Koch 2001; Koch et al. 1998; Koch and Britton 2001), and endurance exercise training in rats (Koch et al. 2013). Selective breeding generally focuses on identified animals that are high responders and/or low responders in an outbred population, and then breeding those animals within trait (i.e., high-with-high, or low-with-low) for multiple generations. This approach has been successful for a mouse model of high activity [> 127 articles since 1998 (Swallow et al. 1998b)], a rat model of high- and low-aerobic capacity [> 138 articles (Koch et al. 1999)], and a rat model of low- and high-physical activity (Roberts et al. 2013, 2014). Most recently, Xu and Garland (2017) demonstrated in four distinct mouse lines selected as high runners, that each line had differing sets of QTLs associated with activity to suggest that each selected line evolved different physiological approaches to wheel-running activity. As powerful as the selective breeding approach is, at its culmination it examines only one or two inbred strains, and thus has limited genomic investigatory capability. Further, it has been suggested that selectively bred models may be prone to “artificial selective sweeps” in which genome sequences in close proximity to phenotypic alleles evolve, leading to expanding and non-informative genomic information (Atanur et al. 2013).

### Recombinant inbred strains

Recombinant inbred strains (RI) are developed from an initial cross of two inbred strains (F_0_) followed by a cross of their offspring (F_1_) and then 20 generations of brother–sister mating to develop new inbred lines (usually 15–35 lines; Zou et al. 2005) to capture genetic variability of the two founder strains (Bailey et al. 1971). The strength of using RI strains is the capability to perform genetic mapping among several traits and across varying environmental conditions. To date, no studies have utilized RI strains to determine or confirm genetic contributors to either endurance or resistance training-induced adaptations. While RI strains present a useful model towards increasing our understanding of the genetic determinants of exercise training adaptations, a weakness of RI strains is the usual small number of RI lines available and potentially limited phenotypic differences between the originating two founder lines since, on average, any pair of classical mouse strains is IBD across ≈ 50% of their genomes (Roberts et al. 2007). This weakness has been overcome by development of the Collaborative Cross (CC) mouse model (see below) where underlying genetic architecture is controlled, yet is more representative of the genomic variability present in humans.

### Consomic and conplastic inbred strains

A consomic mouse model involves a chromosome substitution from one inbred strain to another (http://www.informatics.jax.org/mgihome/nomen/strains.shtml). An obvious advantage of consomic strains is that they can be utilized as an additional confirmatory measure of strain screen analyses that indicated significant QTLs for a specific trait. However, as with other inbred models, the minimal heterogeneous background is a limitation. Conplastic inbred mice were developed with the intention to analyze interactions between mitochondrial and nuclear genomes (http://www.informatics.jax.org/mgihome/nomen/strains.shtml). Conplastic strains are created by backcrossing the nuclear genome from one inbred strain into the cytoplasm of another (http://www.informatics.jax.org/mgihome/nomen/strains.shtml). Currently, the conplastic approach is the only breeding model that provides an opportunity to analyze interactions between the two genomes. Likewise, the heterogeneity that is needed to develop a bidirectional translation approach between animals and humans is limited. Congenic and consomic inbred strains are two additional rodent models (rats and mice) that have been utilized to determine specific gene regions (congenic) and chromosomal (consomic) contribution to specific phenotypes (Matin et al. 1999).

### Collaborative cross mouse model

The CC mice were developed to unravel the genetic architecture of complex traits and behaviors for translation into human models. In short, the CC mice are a large panel of multi-parent RI strains derived from eight founder strains representing the three major mouse subspecies (A/J, C57Bl/6J, 129Sv/ImJ, NOD/LtJ, NZO/H1J, CAST/EiJ, PWK/PhJ, and WSB/EiJ) that capture approximately 90% of the known genetic variation in laboratory mice with the captured variation being randomly distributed across the genome (Roberts et al. 2007; Churchill et al. 2004; Collaborative Cross Consortium 2012). Subsequent characterization has shown that the eight founder lines contributed equally (11.4–13.5%) to the available CC RI lines (Aylor et al. 2011). All current CC mouse lines have been genotyped and their genome sequences imputed (http://csbio.unc.edu/CCstatus/index.py), providing reference to the genetic composition of each mouse. Additionally, CC mice, as opposed to the available inbred mouse and rat strains, were developed using controlled randomization of genetic factors that allows expression of hidden phenotypes (Rogala et al. 2014). Further, F_1_ crosses of CC lines (CC-RIX lines; Zou et al. 2005) have a statistically significant reduction of within-line phenotypic variability relative to their inbred parents (Zou et al. 2005), support the detection of allele-specific expression differences (Crowley et al. 2015), and importantly model the heterozygous structure of the human genome. Thus, the CC-RIX model could present: (1) genome-wide variation so that all components of exercise-associated systems are interrogated (Collaborative Cross Consortium 2012; Threadgill and Churchill 2012); (2) randomization of genetic variation with no minor alleles so that causative relationships can be identified (Collaborative Cross Consortium 2012); (3) sufficient number of lines to power analyses (Zou et al. 2005, 2014); (4) reproducibility, data integration, robust reproduction, and future use in mechanistic studies (http://csbio.unc.edu/CCstatus/index.py); and (5) a unique platform upon which to determine novel gene functions (Rogala et al. 2014; Bottomly et al. 2012; Ferris et al. 2013; Rasmussen et al. 2014).

### Diversity outbred mouse model

Complementing the CC mice is the diversity outbred (DO) mouse breeding system. DO mice are derived from the same eight founder strains of the CC mice; however, the generation of DO mice begins with a randomized and strict breeding scheme of the CC mice so that each DO mouse is genetically unique (Schmidt 2015; Churchill et al. 2012). The DO and CC mouse model schemas can be used in concert with one another to identify and confirm genes governing a specific trait such as with exercise training adaptations. The DO mice, like the CC mice, could be used to translate findings between mice and humans. At this point, however, definitive exercise-related phenotypes must be identified before conducting exercise training studies with DO mice.

We therefore propose that CC and/or DO mouse models be considered in future studies investigating the heritability of exercise training-induced adaptations. While relatively new, the CC model has been successfully used to mimic several human diseases (Bottomly et al. 2012; Ferris et al. 2007) as well as some exercise traits like voluntary wheel-running and energy balance (Mathes et al. 2011). Thus, employing a model like the CC mice, would enable new horizons in the investigation of exercise training adaptations.

The CC (Threadgill and Churchill 2012; Threadgill et al. 2002) and DO (Churchill et al. 2012) mouse models mimic genetic variation in humans (Roberts et al. 2007) and have been used to dissect energy regulation pathways in the healthy condition (Mathes et al. 2011). Some studies suggest translation to humans using the CC mouse model in diseases including influenza (Bottomly et al. 2012; Ferris et al. 2013) and Ebola (Rasmussen et al. 2014). Similarly, the DO mouse model also has suggested translational properties between mice and humans in various drug toxicity-related studies (Harrill 2016; Church et al. 2015), and some have shown that genetically diverse mice can be used to identify phenotypic markers that are unidentifiable in standard inbred mouse models. Thus, CC and/or DO models provide an approach for bidirectional translation between humans and mice, and could be utilized in studies to examine exercise-induced adaptations. Other animal models have been used to investigate exercise-related phenotypes; however, such models pose major limitations that can be overcome with the CC and/or DO mouse models.

## Exercise training-induced inter-individual variation in human and animal models

A weakness in the literature is that animal studies have not been designed to match exercise training designs/responses from human studies. While separate studies in animals and humans provide significant and suggestive genetic factors (QTLs, genes) with exercise training-induced responses, such findings are not translatable between animals and humans given the inconsistencies in training designs and phenotypic measures. Thus, a phenotype must be defined for exercise training-induced adaptations that translate between animal models and humans.

### Endurance exercise training

#### Overview

A wide variety of general systemic and organ responses to endurance training in humans have been well documented over the past 70–80 years. In particular, changes in aerobic capacity (VO_2max_) and extensive changes in metabolic enzymes such as succinate dehydrogenase (SDH), phosphofructokinase (PFK), and hexokinase, along with several other biomarkers, are often used as direct indices of successful adaptation to repeated endurance exercise exposures. Furthermore, numerous structural adaptations in various tissues and organs (e.g., heart, skeletal muscle, vasculature), in addition to functional changes at the cellular and molecular level in systems, respond and adapt to endurance exercise training.

In general, the average change to a treadmill training program in VO_2max_ is approximately 24–25% (Bouchard and Rankinen 2001; Kohrt et al. 1991) in young and older populations without significant influence of weight or sex on the training response. These changes in aerobic capacity are generally indicative of alterations in central (cardiac output) and peripheral (muscle metabolism) factors, and there are well-known alterations in muscle/mitochondrial enzyme levels, capillary density, and increases in ventricular end diastolic volume and contraction strength to increase ventricular stroke volume. At least 50% of these adaptation responses to endurance training are heritable (Bouchard et al. 1999). This genetic influence is clearly shown when considering the broad range of responses to a controlled endurance training program (summarized in Table 1). Thus, it is critical that any attempt to understand the molecular mechanisms responsible for adaptation to endurance training consider underlying genetic architecture so that the widest range of potential causative mechanisms can be captured.

**Table 1:** Summary of endurance training genetic studies in humans and rodents

Study	Species	Subject characteristics	Endurance training study design	Inter-individual/-strain variation in aerobic capacity measures
Bouchard and Rankinen (2001)	Human	HERITAGE Study Cohort: 481 individuals (male = 236; female = 245; 17–65 years of age) from 99 two-generation families from a Caucasian descent	Duration: 22 weeks. Frequency: 3 days/week. Time and intensity: subjects started at 55% for 30 min then progressed to 75% for 50 min by 14 weeks, then maintained until week 20. Type: cycle ergometer	−5–56% increase in VO_2max_
Bouchard et al. (1999)	Human	HERITAGE Study Cohort: 481 individuals (male = 236; female = 245; 17–65 years of age) from 99 two-generation families from a Caucasian descent	Duration: 20 weeks. Frequency: 3 days/week. Time and intensity: from 1 to 14 weeks: 55% initial VO_2max_ for 30 min/day. 14–20 weeks: 75% initial VO_2max_ for 50 min/day. Type: cycle ergometer	The mean increase in VO_2max_ reached ∼400 mL/min. There was considerable heterogeneity in responsiveness, with some individuals experiencing little or no gain, whereas others gained > 1.0 L/min. There was 2.5 times more variance in VO_2max_ between families than within families. Maximal heritability was estimated to be 47% for the VO_2max_ response, which was adjusted for age and sex with a maternal transmission of 28% in one of the models
Kohrt et al. (1991)	Human	Older adults; 300 men and women ages 60–71 years	Duration: 9–12 months. (36–48 weeks). Frequency: 4 days/week. Time and intensity: from 1 to 3 months: 40 min at 75% HR_max_, then progressed to 50 min/day at 75–85% for the rest of the study. Type: walking/running	0–42% increase VO_2max_
Massett and Berk (2005)	Mouse	Male inbred strains (C57BL/6J, FVB/NJ, and BALB/cByJ) and hybrid F_1_ strains: [CB6F1/J (CB6 = female Balb/c × male BL6), B6F F1 (female BL6 × male FVB), and FB6 F1 (female FVB × male BL6)]	Duration: 4 weeks. Frequency: 5 days/week. Time and intensity: 60 min/day, ≈ 60% of max work load. Type: treadmill running	FVB/NJ increased by 160%, C57BL/6J increased by 35%, and Balb/cByJ increased by 21%. FVB/NJ strain increased the distance ran by 2.6-fold. FVB/NJ also had 172% increase in exercise performance, while Balb/cyByJ and C57BL/6J only by 23–33% increase
Kilikevicius et al. (2013)	Mouse	Male A/J, BALB/cByJ, C3H/HeJ, C57BL/6J, DBA/2J, PWD/PhJ, and strains	Duration: 5 weeks. Frequency: 5 days/week. Time and intensity: the time spent swimming was progressively increased from 15 to 150 min/session. Type: swimming	Changes in endurance was strain-dependent where C57BL/6J and DBA/2J improved substantially, and A/J and BALB/cByJ strains did not. Swimming endurance in DBA/2J strain was ~ 9 times better than BALB/cByJ
Massett et al. (2015)	Mouse	Two types of intercross breeding schemes based on strain: (1) male NZW/LacJ and 129S1/SvlmJ mice; and (2) male C57BL/6J and FVB/NJ mice	Duration: 4 weeks. Frequency: 5 days/week. Time and intensity: 60 min/day, ≈ 65% of max work load. Type: treadmill running	FVB/NJ increased by 160%, C57BL/6J increased by 35%, and Balb/cByJ increased by 21%. FVB/NJ strain increased the distance ran by 2.6-fold. FVB/NJ also had 172% increase in exercise performance, while Balb/cyByJ and C57BL/6J only by 23–33% increase
Koch et al. (2005)	Rat	Inbred rat strains previously identified as genetic models of low [Copenhagen (COP)] and high [Dark Agouti (DA)] intrinsic (untrained) exercise capacity	Duration: 8 weeks. Frequency: 5 days/week. Time and intensity: 60 min/day, ≈ 80% of max distance achieved during testing. Type: treadmill running	The low-capacity COP strain increased by 21% and the DA increased distance run by 36%
Koch et al. (2013)	Rat	Males and females; genetically heterogeneous rat population (N/NIH rats)	Duration: 8 weeks. Frequency: 3 days/week. Time and intensity: each session was targeted for a total of 618 min of running time, a total distance of 9,865 m, and a cumulative vertical climb of 2,553 m. Type: treadmill running	Exercise capacity, defined as the change in distance with training, showed inter-individual variation ranging from − 339 to + 627 m

*VO*_*2max*_ maximal aerobic capacity, *HR*_*max*_ heart rate max

#### Human studies

In humans, the HERITAGE (HEalth, RIsk factors, exercise Training And Genetics) study has been the primary source of our current understanding of the inter-individual variation in response to endurance exercise training. The hallmark investigation of the HERITAGE study was a twin study of otherwise healthy untrained male identical brothers (Prudhomme et al. 1983). This study found that significant variations in aerobic capacity improvement between pairs were six to nine times greater than within identical twin pairs. The findings were then replicated in four different centers in the U.S. (Indiana University, University of Minnesota, and Texas A&M University) and Canada (Laval University in Quebec). Each center found a similar spectrum of responses in aerobic capacity improvement, where there remained approximately 15% of individuals who responded very little (non-responders), and then another approximate 15% of individuals who responded well to training (high responders) (Bouchard and Rankinen 2001). Another key finding from the HERITAGE study is that family members generally had similar responses to training, where an individual’s ability to respond to endurance training was approximately 50% heritable. The other 50% of an individual’s ability to respond to training is primarily attributed to unique environmental factors, though age, sex, and ethnic origin are not determinants of the training response (Bouchard and Rankinen 2001). The most recent HERITAGE study (Bouchard et al. 1985) indicated that 21 single nucleotide polymorphisms (SNPs) accounted for 49% of the variation in VO_2max_ with training, where ≤ 9 alleles associated with low responders and ≥ 19 alleles associated with high responders. The strongest associations were found in genes including *ACSL1, PRDM1, GRIN3A, KCNH8*, and *ZIC4* (Bouchard et al. 1985).

Contrary to the conclusions from the HERITAGE study that inherent inter-individual variability exists in response to endurance training, is recent discussion that the observed ‘non-response’ is a result of flawed training designs, where some investigators argue that the “non-response” to training is abolished with increased dose of exercise (duration and intensity of training; Montero and Lundby 2017; Lundby et al. 2016; Bonafiglia et al. 2016). For example, a recent study (Montero and Lundby 2017) conducted two-successive 6-week endurance training programs, where subjects were assigned to one of six groups based on the total minutes of exercise per week: 60, 120, 180, 240, and 300 min/week of exercise. The study concluded that, following the first 6 weeks of training, there were 69, 40, and 29% “non-responders” in each respective training group. However, following a second 6-week training session, no “non-responders” remained suggesting that increasing the dose of exercise for the previously classified “non-responders” may be the remedy to prompt a response to increase aerobic capacity with training. A study by Bonafiglia et al. (2016) supports the claim of abolishing non-responders by increasing training. In this study, it was found through a randomized crossover study design of two training protocol types consisting of endurance training (30 min/day at 65% VO_2peak_) and sprint interval training (eight, 20-s intervals at 170% VO_2peak_ separated by 10-s active rest), the individual response to training was dependent on the training protocol utilized. While there was inter-individual variation within each training protocol, all individuals responded to at least one training protocol to suggest that the “non-response” may be decreased by changing the training stimulus. In totality, these studies suggest that the “non-response” to endurance exercise is abolished through increasing the training stimulus. These findings are interesting, but considerably larger sample sizes are needed to confirm this argument given that there were only 21 and 78 subjects in each study, respectively (Montero and Lundby 2017; Bonafiglia et al. 2016), as well as a lack of diversity of subject background (both sex and ethnicity). Comparatively, the HERITAGE study was conducted across four major institutions and with over 800 individuals representing a wide range of diversity in age, sex, and ethnic background. Thus, the issue of whether “non-responders” exist, when measured by aerobic capacity, to endurance exercise training is not resolved, and deserves further attention.

#### Rodent studies

Endurance training results similar to humans have been observed using mouse models. For example, Courtney and Massett (2012) and Lightfoot et al. (2001) performed strain screens of acute response to endurance exercise in 34 (Courtney and Massett 2012) and 10 (Lightfoot et al. 2001) inbred mouse strains which demonstrate inter-strain variation in exercise capacity. Other studies have reported (Massett et al. 2009, 2015; Massett and Berk 2005) adaptation to endurance training among several inbred strains as well as several F_1_, F_2_, and backcross populations. These results have been expanded recently to include 25 inbred strains, and found a change in endurance capacity after training ranging from − 5.1 ± 3.9% to 20.0 ± 7.3%. Interestingly, when considering the strain screen analysis of inherent endurance exercise capacity by Massett and Berk (2005), the variation in the mouse model is similar to the inter-individual variation in humans (Bouchard et al. 1999). Additionally, when presented as familial data as opposed to individual data, the human familial variation data from Bouchard et al. (1999) and Bouchard and Rankinen (2001) were similar to that found between inbred strains of mice (Massett et al. 2009, 2015; Courtney and Massett 2012; Massett and Berk 2005), with a similar degree of heritability (interclass correlations approximately 65%; coefficient of genetic contribution approximately 47%; Massett and Berk 2005).

The key to successful translatability of results from animal models to humans is to equate the training programs. A weakness of extant rodent literature regarding endurance training is the wide range of training programs used, especially in the duration and intensity of times used. One critical factor listed by the American Physiological Society (Kregel et al. 2006) when designing exercise protocols for animals is animal safety. An ongoing concern is that some protocols (Kemi et al. 2002) may be too long in duration and/or too high in intensity setting leading to less adherence and increased risk of injury and overtraining in the animal subjects. The majority of protocols reported (Massett et al. 2009, 2015; Courtney and Massett 2012; Lightfoot et al. 2001; Massett and Berk 2005; Desai et al. 1997; Petrosino et al. 2016) have intensities and durations more translatable to humans. For example, the training protocols for the mouse endurance model by Massett and colleagues (Massett et al. 2009, 2015; Massett and Berk 2005) were designed to align with the standard, currently recommended human endurance training protocols using a duration of 30 min or more (with 60 min preferred), an intensity between 40–75% VO_2max_, and a frequency of 3–6 days/week (Pescatello and American College of Sports Medicine 2014).

Exercise-induced adaptations with endurance training vary by the modes of training utilized (e.g., treadmill running, swimming, cycling), the length of training time (weeks; months), and the frequency, duration, and intensity of training programs. Exercise training in rodents has generally consisted of methods to induce continuous aerobic exercise. Treadmills, swimming, and running wheels have been used in training studies in rats and mice (Kregel et al. 2006; Kilikevicius et al. 2013). Acute exercise responses in mice on a treadmill are similar to what is seen with humans (Acosta et al. 2017). Given mice are quadrupeds and humans are bipeds, raises the concern whether treadmill training in mice induces physiological adaptations similar to those found in humans. Therefore, some studies have instead used swimming protocols to mimic similar movements. Swimming, however, does not appear to be an adequate model for continuous aerobic exercise in rodents because some work suggests that swimming protocols for rodents causes a series of hypoxic exposures due to floating and bobbing motions by most rodents when they swim (Kregel et al. 2006). With treadmill aerobic capacity testing, Desai et al. (1997) have shown in four inbred and two outbred mouse strains that a linear increase in heart rate, VO_2_, VCO_2_, and RER occurs with an increase in exercise intensity indicative of an escalation in metabolic cost of workload, which is similar to humans. These effects have been observed in several other studies that have measured responses to treadmill running in mice (Fluckey et al. 1996a, b). Thus, while locomotive behavior for running in mice and humans are different, the physiological responses that occur are similar. Therefore, treadmill training is the most applicable mode of training for bidirectional translation of findings between mice and humans.

### Resistance training

#### Overview

A wide literature base exists regarding muscular adaptations from repeated exposure to resistance training in humans. Measures of adaptation in humans involve non-invasive measures including increased strength and muscle size alterations, and direct measures including muscle fiber size changes and contractile mechanism alterations. Additionally, while investigators have long noted inter-individual variation in response to resistance training (Fluckey et al. 1996b), few studies evaluated genetic influence on adaptation to resistance training.

#### Human studies

Thompson et al. (2004) designed the “Functional Single Nucleotide Polymorphisms Associated with Human Muscle Size and Strength” (FAMuSS) study where they examined responses of ≈ 1300 young adults (men and women) to a 12-week, standardized isometric and dynamic (both concentric and eccentric contractions) resistance training program on the non-dominant arm. As part of the FAMuSS study, in 585 subjects (342 women, 243 men), Hubal et al. (2005) found that participants increased mean (± SEM) muscle size by 18.9 ± 0.4%, increased isometric strength by 19.5 ± 0.8%, and increased 1 repetition max (1RM) strength by 54.1 ± 1.4%. Unlike endurance training, sex appeared to affect the amount of change in strength and size (Hubal et al. 2005). Women had greater percentage increases in isometric strength and 1RM strength compared to males, while males increased muscle size significantly more than females. Further, similar to endurance training (Thomis et al. 1998), Hubal et al. (2005) found extensive inter-individual variation in responses to resistance training, with populations of responders and non-responders in each sex for cross-sectional area, 1RM strength gain, and isometric strength. A limitation of this extensive study was its consideration of a fairly homogenous population of relatively young subjects (ages 18–40 years), with primarily European-American backgrounds. However, while FAMuSS may be limited, it is the only existing population-based resistance training study in either humans or animals.

#### Rodent studies

In general, there are fewer models of animal resistance training than endurance training. While agriculture has consistently selectively bred livestock for muscle size (especially work horses and beef cattle) over the past several hundred years, mutations targeted in livestock (e.g., myostatin and IGF-2) have not been found to support any of the suggested QTL from the FAMuSS study (Thompson et al. 2004). This lack of support suggests that muscle size and strength as a result of selective breeding and the size and strength adaptations to resistance exercise exposure potentially arise from different molecular pathways.

While no animal resistance training genetic studies exist, multiple acute resistance training models have been developed for rodents (Kregel et al. 2006; Lowe and Alway 2002; Seo et al. 2014). The primary species in these studies are rats, and outcome variables include measurement of muscle mass, fiber cross-sectional area, contractile function, and fractional protein synthesis rates (Bodine and Baar 2012). The ‘jump-squat’ model of resistance training has been used in several studies (Burgess et al. 1993; Klitgaard 1988; Nicastro et al. 2012; Tamaki et al. 1992; Wirth et al. 2003), and is currently the method of choice for maximal translation to human modes (Kregel et al. 2006; Lowe and Alway 2002). Briefly, the jump-squat protocol in rodents involves operant conditioning to avoid a brief foot shock (< 1 mA, 60 Hz) by extending hind limbs fully to press an illuminated bar repeatedly. After conditioning (3–4 training sessions), rats usually do not need shock to engage in resistance training (Fluckey et al. 1995). Following operant conditioning, animals jump-squat in training sessions with prorated weight vests. Thus, training programs control the amount of resistance and number of reps similar to human resistance training programs. In general, with 8–36 weeks of exposure in rats, there is an average 10–30% increase in muscle mass, a 10–30% increase in cross-sectional area, and a 10–60% increase in force production (Lowe and Alway 2002). These responses are similar to those found in humans by Hubal et al. (2005) in the FAMuSS study. Furthermore, rates of protein synthesis are significantly increased in both gastrocnemius and soleus muscle with either short-term (four training session in young, middle-aged, and old rats; Fluckey et al. 1996a) or long-term (8 weeks of training; Farrell et al. 1999) exposures.

These studies suggest that weight-lifting types of exercise are analogous to human resistance training programs and have found similar muscle-physiological adaptation compared to human programs. A weakness of weight-lifting types of exercise is that the exercise is bilateral, removing the ability to have an internal control (Watt et al. 1982), as present in FAMuSS (Hubal et al. 2005). This weakness could be overcome using CC and/or DO mouse models that control for the underlying genetic architecture so that independent control animals can be used that do not undergo resistance training. Lastly, while rodent models can mimic general human muscle adaptation to repeated resistance training programs, a weakness of most of these studies is that they exclusively used male animals. Thus, future research utilizing both sexes is needed.

## Potential role of the mitochondrial genome and its interactions with the nuclear genome

### Overview

To enable a complete understanding for how genetic background contributes to exercise training adaptations, it is important to also consider the role of the mitochondrial genome. Few studies have investigated links between the mitochondrial genome and endurance training adaptations (Dionne et al. 1991; Rivera et al. 1998; Lihong 2000; Chen et al. 2000; Bray et al. 2009). However, conclusive findings from such studies are limited because they focus only on certain portions of the mitochondrial genome. Recent advances in technologies, including Next-generation Sequencing, have enabled complete and ultra-deep sequencing of mitochondrial DNA in animal and human tissues that contain mitochondria. Furthermore, ultra-deep sequencing capabilities have enabled discovery of other important elements of the mitochondrial genome, including heteroplasmy and insertions/deletion (indels) that could also have significant roles in acute exercise and adaptations to training. To date, the role of the mitochondrial genome and its interactive effects with the nuclear genome on adaptive phenotypes of exercise training are not fully understood. The mitochondrial genome also presents an additional level of complexity due to cell-to-cell variation in mitochondrial DNA copy number that is dependent upon the energy requirement of a cell (Taylor and Turnbull 2005). Since relatively new techniques have allowed for accurate measurement of mitochondrial DNA copy number and heteroplasmy, such factors have not been fully explored. Taken together, studies are needed to assess their association with inherent exercise training adaptations in endurance and resistance training.

Briefly, mtDNA is circular and spans approximately 16,500 DNA-building base pairs that make up 37 genes: 13 protein-coding genes provide instructions for making enzymes involved in oxidative phosphorylation, 2 transfer RNAs (tRNA), and 22 ribosomal RNAs (rRNA) (Chinnery and Hudson 2013; Schon et al. 2012). One unique characteristic of the mitochondrial genome compared to the nuclear genome is that few to hundreds of thousands of mtDNA copies may exist per mitochondrion. With multiple copies of mtDNA within mitochondria, sequence variants can result due to various factors such as maternal mutation(s) passed to offspring, age, diseases, and other environmental exposures. Such variants in mtDNA lead to a heterogeneous population of mtDNA called heteroplasmy. To date, no study has considered the contribution of mtDNA copy number and/or heteroplasmy as it relates to exercise training-induced adaptations.

The mitochondrial genome is of particular interest with regard to exercise training adaptations because of the crucial role of mitochondrial DNA in regulating overall mitochondrial function. A major function of the mitochondrial genome is to regulate mitochondrial function [the production of energy in the useful form of Adenosine Triphosphate (ATP), and also respond to production of reactive oxygen species (Voet et al. 2013)]. There are also approximately 1,300 nuclear genes that interact with mtDNA and encode mitochondrial proteins (Andersson et al. 2003). Thus, mitochondrial function is dependent on mitochondrial and nuclear DNA interactions. With exercise training, the mitochondrial responses are critical to enable increased aerobic capacity as well as increased muscle strength and size with endurance (Brearley and Zhou 2001) and resistance training (Huffman et al. 2014), respectively. The inability of the mitochondria to respond and adapt in a positive manner to exercise training would inhibit an individual from responding to exercise, and in some cases, potentially decrease the ability to respond to exercise if mitochondrial damage ensued. One major source of mitochondrial DNA damage is through production of reactive oxygen species. With endurance training, aerobic capacity (VO_2max_)—the primary indicator of how well individuals respond to endurance training—is partly dependent upon how well the mitochondria respond to exercise-induced reactive oxygen species production, and their adaptability to increase efficiency of ATP production (e.g., induce mitochondrial biogenesis). Functions and adaptations of the mitochondria are heavily dependent upon the mitochondrial genome. Because mitochondrial DNA regulates overall mitochondrial function, and mitochondrial function significantly contributes to an individual’s aerobic capacity, investigators have hypothesized that the mitochondrial DNA sequence is associated with the inherent ability to respond to exercise training (Dionne et al. 1991; Scott et al. 2005). In fact, in the 2006–2007 update of the “Human Gene Map for Performance and Health-Related Phenotypes” (Bray et al. 2009), 18 mitochondrial genes were shown across various studies to influence fitness and performance phenotypes.

### Mitochondrial DNA sequence variants

Several mitochondrial DNA sequence variants have been associated with exercise intolerance (Hirano et al. 2001; Goethem et al. 2001; Zeviani et al. 1989; Wallace 1992). However, most studies focused on individuals with a mitochondrial DNA mutation-induced diseases that severely inhibit exercise. Such studies may provide insight into specific locations of the mitochondrial genome that influence an individual’s response to exercise training. Using the restriction fragment length polymorphism (RFLP) technique, Dionne et al. (1991) compared mtDNA sequence polymorphisms in the untrained and trained states. Of the mtDNA variants considered, the authors found the subunit 5 of the NADH dehydrogenase gene and one in the tRNA for threonine, had a significantly higher VO_2max_ in the untrained state when compared to non-carriers, while carriers of one mitochondrial DNA morph in subunit 2 of NADH dehydrogenase had a lower initial VO_2max_. A lower response to endurance training via cycle ergometry was found for three carriers of a variant in subunit five of the NADH dehydrogenase. Thus, while the RFLP technique is limited in terms of fully uncovering the contribution of mtDNA sequence and polymorphisms to the inter-individual responses to exercise training, this study did indicate that the mitochondria genome may have a role in determining VO_2max_ responses which warrants further investigation. Challenging this previous finding, Scott et al. (2005) considered mtDNA haplogroups in a general Ethiopian population and an elite athlete runner Ethiopian population. The study found that the groups did not differ in terms of their mtDNA haplogroup, and therefore hypothesized that running ability was not determined by mitochondrial DNA polymorphisms.

While mitochondrial DNA haplogroups did not associate with exercise training adaptations in this study, there are other important elements of the mitochondrial genome that should be considered, including mtDNA copy number. At the cellular level, mitochondrial biogenesis is a key contributor to increased aerobic capacity with exercise training, and mtDNA copy number has been shown to increase concomitantly with exercise-induced mitochondrial biogenesis occurring in the skeletal muscle with exercise training (Puntschart et al. 1995; Trounce 2000). Thus, there could be significant influential effects of mtDNA replication in each mitochondrion that initiate mitochondrial biogenesis.

There are, however, several studies that support the notion that the mitochondrial genome significantly contributes to inherent exercise capacity (Chow et al. 2007; Robinson et al. 2017). Chow et al. (2007) demonstrated that aerobic exercise training enhances muscle mitochondrial transcription factors, mtDNA abundance, mitochondria-related gene transcript levels, and mitochondrial function. Additionally, the authors suggested that the enhancement in mitochondrial function occurs in association with increased spontaneous physical activity (Chow et al. 2007). Robinson et al. (2017) examined different types of exercise training, and demonstrated an effect of intensity on mitochondrial respiration. When comparing continuous aerobic training to “high intensity interval training” (HIIT), it was found that HIIT induced the greatest changes in gene expression and mitochondrial respiration. Resistance training was also examined, and unlike the endurance exercise training protocols, it did not induce any changes to factors related to mitochondrial function or amount. Another study by Robinson et al. (2017) found after 12 weeks of resistance training, young and old individuals had no changes in mitochondrial function measurements, which also indicated that the mitochondrial genome is unlikely involved with the resulting training adaptations. The authors further investigated factors related to intrinsic mitochondrial function including coupling efficiency and reactive oxygen species production, as well as mitochondrial protein abundance, and the only change with training was mitochondrial protein abundance. That is, the amount and not function, of the mitochondria is a primary contributor to the exercise endurance training response. Thus, from the limited data available on the relationship between genetics and resistance training, it appears that the adaptations to resistance training are independent of the mitochondrial genome.

### Protein 53 (p53)

The tumor-suppressor protein, protein 53 (p53) has a role in mitochondrial function and may determine an individual’s response to exercise training. p53 is encoded by the *Trp53* gene in mice and *TP53* gene in humans, and contributes to inherent aerobic capacity (Bouchard et al. 2015). p53 can regulate mitochondrial function through its role as a nuclear transcription factor, and also through its direct presence in mitochondria via translocation. Bouchard et al. (2015) reviewed such work describing pathways in which p53 participates in mitochondrial biogenesis and thus directly affecting exercise capacity. An important finding about p53, particularly when considering the potential role that the mitochondrial genome may play in determining exercise capacity, is that absence of p53 in mice (p53 knockout mice) results in significant depletion of mtDNA and consequently poor exercise capacity, both at baseline and with training (Matoba et al. 2006; Park et al. 2016; Saleem et al. 2009). In humans, p53 also influences recovery time with exercise. A study by Wang et al. (2013) showed that in individuals with the Li–Fraumeni syndrome, which have a mutation in the *TP53* gene, had significantly faster recovery than non-carrier and healthy controls from a 2-min bout of exercise when assessed by phosphocreatine levels in skeletal muscle after exercise. While such studies suggest that genetic alteration to the *Trp53* (mice) and *TP53* (humans) gene has a necessary role in regulating responses to exercise training through its association with mitochondrial biogenesis and respiration, the precise mechanism through which this occurs is not fully understood (Bouchard et al. 2015).

Another important role of p53 in response to endurance exercise-induced training is to regulate mitochondrial polymerase gamma (POLG1). POLG1 is an enzyme considered to be the only mtDNA repair enzyme, and has been demonstrated to initiate mtDNA mutation repair and mitochondrial biogenesis in response to endurance exercise training (Safdar et al. 2016). By inducing skeletal muscle-specific deletion of p53 in mice, Safdar et al. (2016) showed that endurance training in these mice failed to prevent mtDNA mutations, induced mitochondrial biogenesis, preserved mitochondrial morphology, reversed sarcopenia, or mitigated premature mortality, when compared to controls. From the series of experiments by Safdar et al. (2016), p53 was shown to have a direct role on mtDNA mutation repair and induction of mitochondrial biogenesis. Taken together, the interaction between p53 and exercise capacity highlights a nuclear gene that not only has a direct influence on exercise capacity, but also provides a direct mechanistic pathway through which both the nuclear and mitochondrial genomes associate with exercise capacity.

Bioinformatic and statistical methods are still needed to analyze and determine the interactive and solitary effects of mitochondrial and nuclear genome associations with specific phenotypes, though there are available methods in mice that enable insight into these effects. As noted earlier, conplastic strains have been used to determine the contribution of the mitochondrial genome and its interactions with the nuclear genome, in a variety of phenotypes (http://www.informatics.jax.org/mgihome/nomen/strains.shtml). Another animal, known as the mitochondrial–nuclear eXchange Mice, is a model that allows the combination of the nuclear genome from one mouse strain, via oocyte enucleation, with mtDNA from a different mouse strain, a method similar to the well-known ‘3-parent babies’ (Kesterson et al. 2016). The mitochondrial–nuclear eXchange mice would be particularly interesting to use in mice previously characterized as having high, low, no response, and lowered responses to exercise training (e.g., Massett and Berk 2005; Kilikevicius et al. 2013). For example, determining the effect of mtDNA of a high responder strain on the nuclear genomic background of a low responder strain to exercise, and vice versa. Together with the recent techniques such as next-generation ultra-deep sequencing, such methods have enabled multiple avenues for future research regarding interactions between the mitochondrial and genomes that may associate with exercise training-induced phenotypes.

## Conclusions and future directions

The preponderance of evidence that demonstrates an existing association between genetics and physical activity is drawn primarily from considering the nuclear genome while few efforts have considered the mitochondrial genome. From the human and mouse studies by Bouchard et al. (1999) and Massett et al. (2009, 2015), and Massett and Berk (2005), the function and proliferative response of mitochondria (i.e., surface area; density) in energy-demanding tissues (e.g., heart, skeletal muscle) are imperative to enable significant exercise training responses (e.g., increased VO_2max_). However, attention is yet to be directed on how the mitochondrial genome influences such adaptations. Furthermore, given that genetic background strongly contributes to exercise training-induced responses, and that other lines of work hint towards a significant contribution of the mitochondrial genome (e.g., mediation by p53; Safdar et al. 2016), it is of interest to know whether mitochondrial–nuclear interactions exist to predict mitochondrial adaptations/responses to exercise training.

In conclusion, we suggest that future work investigating the relationship between exercise and genetics consider employing animal models such as the CC or DO mice that are designed to control for the genetic diversity inherent in human models (Roberts et al. 2007; Threadgill and Churchill 2012; Threadgill et al. 2002, 2011). Given that such controlled models are already being successfully used to understand the genetic foundation of several human diseases (Bottomly et al. 2012; Ferris et al. 2013) as well as some exercise traits like voluntary wheel-running and energy balance (Mathes et al. 2011), the use of these models will enable new horizons in the investigation of exercise training adaptations. Furthermore, the CC animal modeling scheme could also be used to delineate the contribution of the mitochondrial genome and its interactive effects with the nuclear genome to exercise responses, given that the breeding strategy is designed in a way to determine solitary and combined associations with each genome.
